# Synergistic Effects of Radiotherapy and PD‑1 Blockade in a Human‑Mimetic BRCAness Model of Triple-Negative Breast Cancer

**DOI:** 10.7150/ijbs.118427

**Published:** 2025-10-01

**Authors:** Eun Ju Cho, Min Kyung Ki, Hye Jung Baek, Dong Hoon Shin, Eun Jung Park, Tae Hyun Kim, Chu-Xia Deng, Beom K. Choi, Sang Soo Kim

**Affiliations:** 1 Research Institute, National Cancer Center Research Institute, Goyang, 10408, Korea.; 2 Cancer Centre, Faculty of Health Sciences, University of Macau, Macau SAR 999078, China.; 3 Innobationbio, Co., Ltd., Mapo-gu, Seoul, 03929, Republic of Korea.

**Keywords:** BRCA1, breast cancer, immune checkpoint, PD-1, irradiation

## Abstract

BRCA1‑deficient triple‑negative breast cancer (TNBC) presents significant treatment challenges owing to the absence of estrogen receptor (ER), progesterone receptor (PR), and human epidermal growth factor receptor 2 (HER2) targets, exhibits marked molecular heterogeneity that precludes the application of effective targeted therapies, and harbors a highly immunosuppressive tumor microenvironment. Here, we used the *Brca1^co/co^ MMTV‑Cre* mouse model that recapitulates human *BRCA1*‑mutant TNBC, characterized by early dominance of CD11b⁺Gr‑1⁻F4/80^Low^ blood‑derived macrophages and subsequent enrichment of F4/80^High^ tissue macrophages within adipose‑rich mammary glands. PD‑1 blockade with anti‑mPD‑1 monoclonal antibodies (mAb) significantly delayed primary tumor progression, reduced proliferation marker levels (PCNA, Ki‑67), enhanced apoptosis (as indicated by increased cleaved PARP levels), and selectively impaired PI3K/AKT signaling. In a post‑resection setting, anti-mPD-1 treatment extended recurrence‑free survival rates, with elevated CD4, CD8α, and cleaved PARP levels observed in recurrent tumors. Mice with the longest relapse‑free intervals exhibited the strongest T cell marker expression. A combination of focal 20 Gy irradiation and PD-1 blockade exerted a potent synergistic effect. Specifically, irradiation reduced extracellular matrix deposition and enhanced tumor cell apoptosis (evidenced by increased cleaved caspase-3 and cytosolic PCNA) while PD-1 blockade stimulated robust inflammatory responses, in particular, expansion of CD8α⁺ T cell infiltration. These mechanistic insights align with clinical strategies for TNBC that integrate DNA damaging agents and immunotherapy and validate this model as an optimal *in vivo* platform for preclinical evaluation of novel treatment modalities for *BRCA1*‑associated breast cancer.

## Introduction

Breast cancer type 1 susceptibility protein (BRCA1) functions as a tumor suppressor essential for maintaining genomic integrity by coordinating several key cellular processes involved in genetic stability, such as DNA damage repair, cell cycle control, centrosome duplication, and apoptosis 1. Germline mutations in *BRCA1* account for a substantial proportion of hereditary breast and ovarian cancers 2. Women with germline *BRCA1* mutations have a 57% (95% confidence interval [CI], 47-66%) risk of developing breast cancer and 40% (95% CI, 35-46%) risk of developing ovarian cancer by 70 years of age 3. Gene and protein expression profiling experiments have demonstrated that tumors arising from *BRCA1* mutations typically exhibit triple-negative and basal-like characteristics, tend to be aggressive, and are generally associated with poor prognostic outcomes 4. Given the high incidence of tumors in *BRCA1* mutant carriers, the National Comprehensive Cancer Network (NCCN) recommends that women with *BRCA1* mutations undergo regular breast screening and consider risk-reducing surgery to lower the incidence of breast cancer (NCCN Guidelines, 2022). The primary treatment option for emerging tumors is surgical resection followed by adjuvant chemotherapy. More recently, adjuvant treatment with Olaparib (AZD2281) has been introduced as an additional viable option for patients with germline *BRCA1* mutations following completion of adjuvant chemotherapy. Although effective therapies for BRCA1-deficient breast cancer are urgently needed, treatment options remain limited at present.

Loss of BRCA1 is associated with the onset of breast cancer and alterations in the immune system within breast tissue are considered to play an important role in this process. The breast immune microenvironment performs several vital functions, including protection of newborns through breast milk, preventing infections in the mammary gland during lactation, and conducting immunosurveillance to eliminate tumor epithelial cells 5, 6. The breast undergoes marked histological changes during puberty, pregnancy, lactation, postpartum involution, and age-related lobular involution 7, 8, which are accompanied by alterations in immune cell composition that could influence the likelihood of developing breast cancer 9-11. Recent studies have shown that alterations in DNA repair pathways can potentially promote susceptibility to immune checkpoint inhibition (ICI) by inducing an increase in tumor mutational burden and neoantigen production 12. However, the precise mechanisms and roles of DNA repair pathway alterations as predictive biomarkers for immunotherapy responses remain to be clarified. *BRCA1* is a key gene involved in DNA repair 13 and its deletion is implicated in hereditary breast cancer. Accordingly, we focused on the relationship between breast cancer arising from BRCA1 loss and increased tumor mutational burden, with the aim of assessing its potential as a predictive indicator for improved outcomes with immune checkpoint inhibitor (ICI) therapy.

In the current study, the antitumor efficacy of current clinical interventions for TNBC was evaluated using an autochthonous *Brca1*-mutant mouse model that accurately mimics the characteristics of human BRCA1-deficient mammary tumors. Given the recent approval of PD-1/PD-L1 immune checkpoint blockade (ICB) for various cancer types 14, we examined the efficacy of anti-mouse PD-1 mAb in this model to determine its ability to enhance T cell-mediated immunity, an effect clinically reflected by higher densities of PD-1⁺ tumor-infiltrating lymphocytes and PD-L1⁺ tumors associated with improved patient outcomes 15, 16. *In vivo* monitoring of tumor progression and relapse revealed that PD-1 blockade effectively suppressed tumor growth, delayed recurrence, and facilitated immune remodeling akin to the responses observed in TNBC patients. The collective results highlight the strong translational fidelity of the *Brca1* mutant model, supporting its utility as an optimal preclinical platform for the evaluation of novel therapeutic strategies for TNBC.

## Materials and Methods

### Animal experiments

*Brca1* conditional-knockout (*Brca1-co*) and *MMTV-Cre* transgenic mice, sourced from the National Cancer Institute (NCI, USA) mouse repository, were originally generated by Dr. Deng and Dr. Hennighausen, respectively 14, 15. All procedures involving animals and their care were approved by the Institutional Animal Care and Use Committee of the National Cancer Center (Goyang, Korea). Female *Brca1* mutant mice were generated by intercrossing *Brca1* conditional knockout and *MMTV-Cre* mice. Genotyping of mice carrying mutant alleles was conducted via polymerase chain reaction (PCR) using previously described 16.

Female *Brca1*-mutant mice (*Brca1^co/co^MMTV-Cre*) were monitored weekly for tumor development, beginning at 8 months of age. Once a mammary tumor reached a volume of ~ 0.5 cm³, animals were randomly assigned to receive either a rat IgG2a isotype control or anti-mPD-1 mAb (RMP1-14 clone, 10 mg/kg, i.p., once weekly) purchased from BioXCell (Lebanon, NH, USA). For adjuvant therapy studies, tumors were surgically resected under the influence of alfaxalone (80 mg/kg, i.p.) combined with xylazine (10 mg/kg, i.p.) anesthesia once volumes reached ~1,000 mm³. Following the surgical procedure, mice were allowed to recover under a heated infrared lamp and administered enrofloxacin (100 mg/L in drinking water) for one week. At one week post-recovery, animals were randomly assigned to receive isotype control or anti-mPD-1 mAb (10 mg/kg, i.p., once weekly). Time to recurrence was defined as the interval (in days) from surgical resection to initial detection of tumor regrowth. When mice developed tumors in more than one mammary gland, we selected the first identified tumor for further analysis. In addition, mice with spontaneously developed mammary tumors did not exhibit evidence of tumor spread to distant organs beyond the mammary glands.

For self-engraftment experiments, mammary tumors were harvested from *Brca1^co/co^MMTV-Cre* mice under alfaxalone anesthesia, as above, and trimmed into uniform 5 mm cubes using a sterile tissue slicer. Two tumor fragments were implanted back into the tumor-containing mouse, one in each of the fourth mammary fat pads located on the dorsal flanks, and secured with wound clips. Mice were allowed to recover under a heated infrared lamp and administered enrofloxacin (100 mg/L in drinking water) for one week. After two weekly treatments with anti-mPD-1 mAb or isotype control, one engrafted tumor per mouse was shielded with a lead plate, while the contralateral tumor was exposed to a single dose of 20 Gy X-ray irradiation delivered via a 225 kV Xstrahl accelerator (30 cm source-surface distance, 1 cm circular collimator, 2.5 Gy/min, XenX; Xstrahl, Camberley, England). During the irradiation process, mice were anesthetized and immobilized on a custom stage. No serious adverse events, including weight loss, were recorded.

Tumor progression was monitored on a biweekly basis through caliper measurements from the onset of treatment. Tumor volume (mm³) was calculated using the formula: V = 0.5 × d² × D, whereby d and D represent the short and long diameters, respectively. Relative tumor volume (RTV) was defined as the volume at each time-point divided by the baseline volume at treatment initiation. Measurements were continued until tumors reached a volume of ~3,000 mm³. No severe adverse events, including significant weight loss, were observed throughout the study.

### Immunoblotting

Tumor tissue lysates were prepared using an electric homogenizer for 30 seconds after the addition of lysis buffer, as described previously 17. Western blot analysis was conducted in accordance with standard procedures using enhanced chemiluminescence detection (GE Life Sciences, Chicago, IL, USA). The following antibodies were used: anti-β-actin, anti-phospho-AKT, anti-phospho-ATM, anti-caspase-3, anti-cleaved caspase-3, anti-cleaved caspase-7, anti-CD4, anti-CD8α, anti-CD11c, anti-CD19, anti-F4/80, anti-phospho-MAPK, anti-PARP, anti-cleaved PARP, anti-mPD-1, anti-PD-L1, anti-phospho-S6, anti-phospho-Rb (Cell Signaling Technology, Danvers, MA, USA), anti-Ki67 (Novus Biochemicals, Littleton, CO, USA), anti-p53 (Leica Biosystems, Nussloch, Germany), and anti-PCNA (Atlas Antibodies, Bromma, Sweden). Horseradish peroxidase-conjugated goat anti-rabbit and mouse antibodies (Jackson ImmunoResearch, West Grove, PA, USA) were used as the secondary antibodies, as appropriate. Relative band intensities were quantified with ImageJ and normalized to β-actin.

### Histology and immunohistochemical staining

For histological analysis, tissues were fixed in 10% (v/v) formalin, embedded in paraffin, sliced into sections, stained with hematoxylin and eosin (H&E), and examined via light microscopy. Immunoreactive proteins were detected using following primary antibodies: anti-cleaved caspase-3, anti-CD4, anti-CD8α, anti-FoxP3, anti-F4/80, anti-mPD-1 (all from Cell Signaling Technology), and anti-PCNA (Atlas Antibodies). Tissues were further stained using a SPlink detection bulk kit (GBI labs Mukilteo, WA, USA) in accordance with the manufacturer's instructions. Terminal deoxynucleotidyltransferase-mediated dUTP-biotin nick-end labeling (TUNEL) assay for identifying apoptotic cells in tissue sections was conducted in line with the supplier's recommendations (Millipore Burlington, MA, USA). Masson's trichrome staining kit was employed to monitor collagen accumulation in tissues (Sigma-Aldrich, St. Louis, MO, USA).

### Flow cytometry

Excised tumors were minced into ~3-5 mm fragments and enzymatically digested in RPMI-1640 containing 0.1% collagenase IV (Sigma-Aldrich) and 100 U/mL DNase I (Sigma-Aldrich) for 30 min at 37°C with gentle agitation. The resulting cell suspension was filtered through a 70 µm mesh, washed with PBS, and resuspended in FACS buffer (PBS + 1% FBS). Cells were incubated with anti-mouse CD16/32 (Fc block) for 10 min at 4°C, followed by staining for 30 min at 4°C with the following fluorochrome-conjugated antibodies: FITC-anti-CD4, PE-anti-mPD-1, and PE-anti-F4/80 (Thermo Fisher, Waltham, MA, USA), PE/Cy5-anti-CD8α and APC-anti-CD45 (BioLegend, San Diego, CA, USA), and FITC-anti-Gr-1 and PerCP-Cy5.5-anti-CD11b (BD Biosciences, Franklin Lakes, NJ, USA). After washing with Stain buffer (BD Pharmingen, San Jose, CA, USA), samples were resuspended in FACS buffer containing propidium iodide (1 µg/mL) to exclude dead cells. Data were acquired using a FACSCalibur (BD Biosciences) and subsequently analyzed with FlowJo software.

## Results

### Tumor localization and immune landscape in *Brca1^co/co^MMTV-Cre* mice

**Figure 1:**
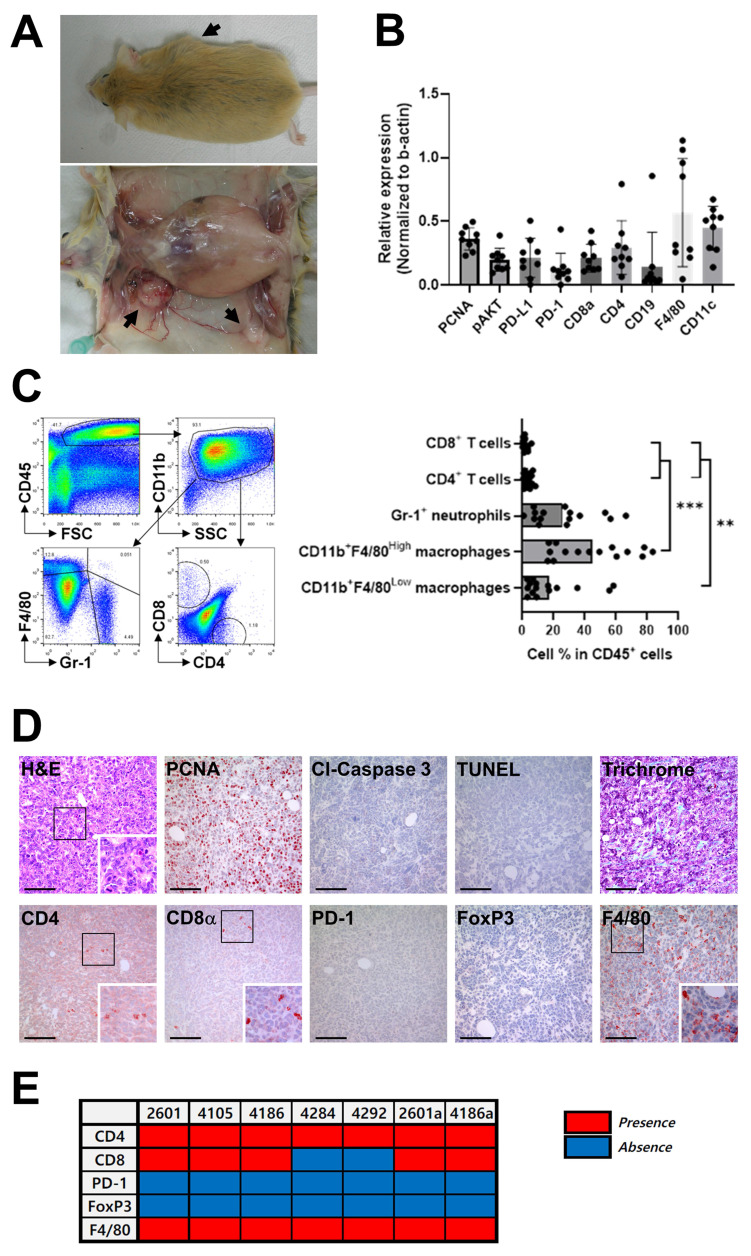
** Immunological characteristics of mammary tumors in *Brca1^co/co^MMTV-Cre* mice.** (A) Representative images of a *Brca1^co/co^MMTV-Cre* mouse showing spontaneous formation of mammary tumors (arrows). The lower panel depicts the excised tumor with visible abnormalities. (B) Western blot analysis of the expression patterns of immune-related proteins in mammary tumors. Band densities were quantified using ImageJ and the relative expression of each antigen determined for each sample by dividing its band density by the corresponding β‑actin band density. (C) Representative flow cytometry plots showing the immune cell composition within a mammary tumor. Myeloid cells were analyzed by initial gating on CD45⁺ leukocytes followed by CD11b⁺ cells, and subsequent plotting of F4/80 vs. Gr-1. Neutrophils were identified as F4/80⁻Gr-1⁺, tissue macrophages as CD11b^+^F4/80^High^, and blood-originated macrophages as CD11b^+^F4/80^Low^. T cells were analyzed by plotting CD8α vs. CD4 within the CD45⁺ leukocyte population. The bar graphs summarize the immune cell subset distribution in tumors collected from 15 individual mice, with one tumor analyzed per mouse. Data are shown as means ± SDs. *p*-values were calculated using Student's t test (**p < 0.01; ***p < 0.005). (D) Histological and immunohistochemical analyses of mammary tumor sections, including H&E, PCNA, cleaved caspase-3, TUNEL, and trichrome staining, as well as immunostaining for CD4, CD8α, PD-1, FoxP3, and F4/80. The boxed areas in the images are enlarged in the lower right. Scale bar: 100 μm. (E) Presence (red) or absence (blue) of the indicated immune cell markers across tumor samples. For each mouse, a single representative tumor was analyzed in panels (B)-(E). The numbers above each lane (B) or in the panel (E) indicate individual mouse identification numbers.

Female *Brca1* conditional knockout mice were generated via *MMTV-Cre*-mediated recombination (*Brca1^co/co^MMTV-Cre*). This model was specifically designed to recapitulate the “BRCAness” phenotype, characterized by BRCA1 deficiency associated with alterations in key cancer-related genes, that exhibits features similar to human triple-negative breast cancer (TNBC), including high-grade histological patterns and basal-like properties 20, 21. While the tumor sites varied among individual mice, a notable increase in mammary tumor incidence was evident by 8 months of age, and by 14 months, over 70% of *Brca1^co/co^MMTV-Cre* mice had developed tumors (Fig. 1A). In contrast, no tumors were detected in *Brca1^co/co^* mice possessing intact BRCA1 (data not shown). Immunological characteristics of the tumors were evaluated via western blot analysis of nine breast cancer tissues from* Brca1^co/co^MMTV-Cre* mice. Robust expression of F4/80, a marker for mouse macrophages, and CD11c, a transmembrane glycoprotein highly expressed by dendritic and Langerhans cells, was observed in all tumor samples (Fig. 1B and Supplementary Fig. 1A). On the other hand, CD19, a marker for B cell development, was detected at low levels in a limited number of tumors (Fig. 1B). Furthermore, T cell markers (CD4 and CD8α) were present at relatively high levels, with both PD-1 and PD-L1 being readily detectable (Fig. 1B). While the absolute levels of PD-1 and PD-L1 varied between individual tumors, the relative proportion of these molecules was consistently maintained within each tumor (Fig. 1B), suggesting coordinated regulation of these immune checkpoints 23, 24. Elevated expression of pAKT and PCNA further indicated continuous rapid proliferation of tumor cells (Fig. 1B). Flow cytometric analysis of single-cell suspensions from tumors revealed that CD45⁺ leukocytes constituted ~40-50% of total cells, with CD11b^⁺^ myeloid cells representing the predominant subset (Fig. 1C). Within the CD11b^⁺^Gr-1^⁻^ macrophage population, the proportions of F4/80^High^ (tissue-resident) and F4/80^Low^ (blood-derived) subsets varied depending on tumor size. This distinction was further supported by analysis of CD11b versus F4/80 expression profiles, which revealed two discrete populations: CD11b^High^F4/80^Low^ cells corresponding to blood-derived macrophages and CD11b^Low^F4/80^High^ cells representing tissue-resident macrophages (Supplementary Fig. 2). A summary graph from 15 mice confirmed the dominance of myeloid cells over T cells, suggesting a myeloid-rich, T cell-deficient tumor microenvironment that could facilitate tumor progression 18. In contrast, the proportions of CD4^+^ T and CD8α^+^ T cells within the tumor were extremely low (< 1%) (Fig. 1C). Immunohistochemical (IHC) analysis of mammary tumor sections from *Brca1^co/co^MMTV-Cre* mice validated our western blot and FACS findings. PCNA staining revealed a high proliferation rate in tumors, accompanied by abundant infiltration of F4/80^+^ macrophages (Fig. 1D). In contrast, CD4^+^ T and CD8α^+^ T cells were sparsely distributed and PD-1- or FoxP3-positive T cells were rarely detected (Fig. 1D). PD-1-positive cells were detected at low frequency within the tumor tissue (Fig. 1D). However, higher-magnification images revealed that these PD-1^+^ cells are scattered immune cells, most likely lymphocytes or macrophages (Supplementary Fig. 3). An overview of the immune cell profiles across seven tumor samples further confirmed consistent F4/80 and CD4 signals, with PD-1 and FoxP3 detected only in a subset of tumors (Fig. 1E). These findings indicate that the PD-1 signals detected by Western blot primarily originate from macrophages rather than tumor cells.

In summary, the *Brca1^co/co^MMTV-Cre* mouse model accurately mimics key characteristics of human TNBC, including tumor localization in fat-rich mammary glands and highly aggressive tumor biology. A notable feature of the model is the prominent infiltration of blood-derived macrophages, which may play a critical role in driving the rapid progression of these tumors 19, 20. The collective findings of this in-depth characterization provide a strong rationale for further exploring immunotherapeutic strategies targeting *BRCA1*-associated breast cancer.

### PD-1 blockade reduces tumor progression and promotes immune infiltration in *Brca1^co/co^MMTV-Cre* mice

**Figure 2:**
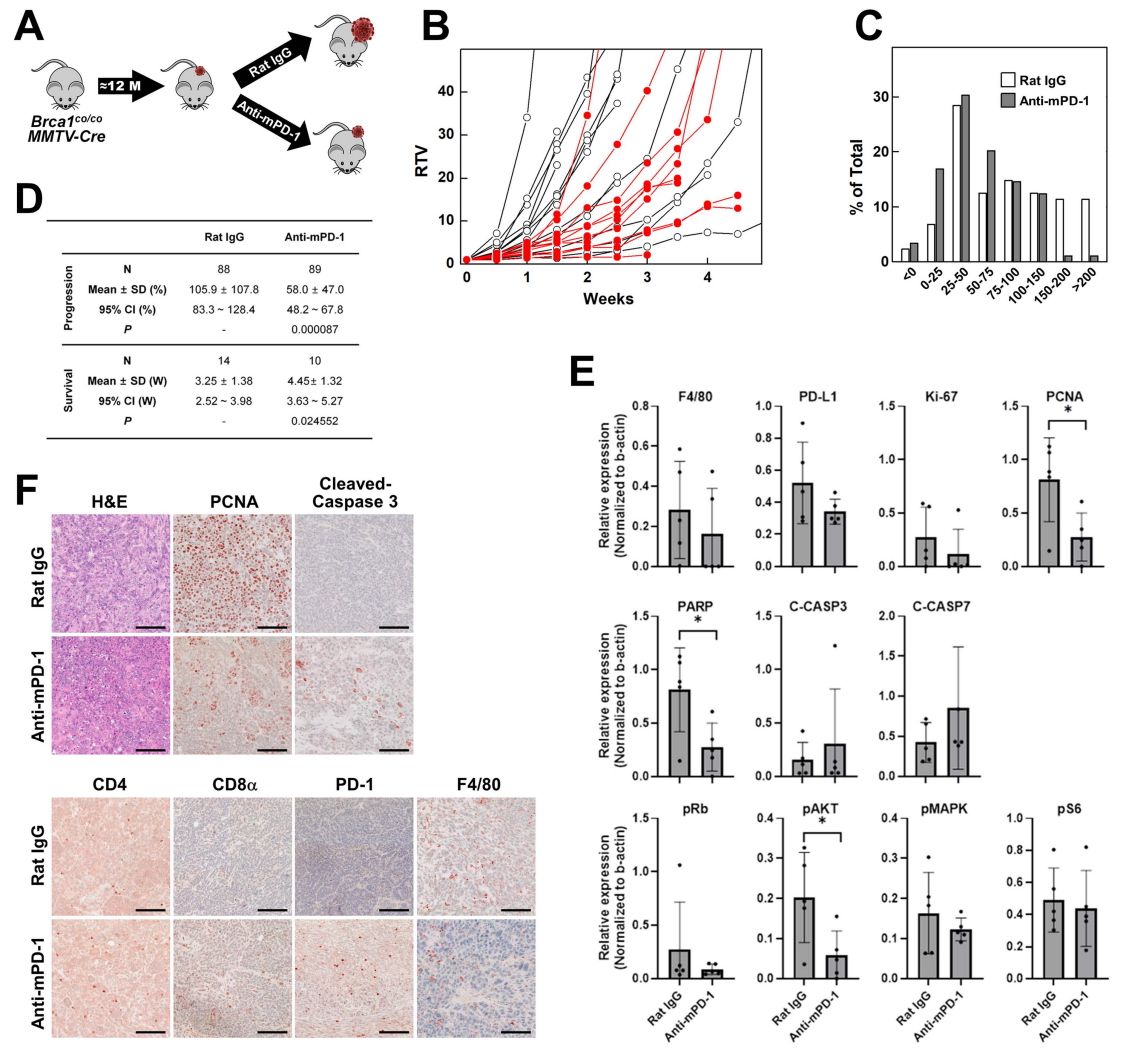
** Therapeutic effects of anti-mouse PD-1 blocking mAb on the progression of mammary tumors in *Brca1^co/co^MMTV-****C**re*** **mice.** (A) Schematic illustration of the experimental design for assessment of anti-mouse PD-1 therapy in spontaneously developed mammary tumors of female *Brca1^co/co^MMTV-Cre* mice (≥10 months old). Upon tumor appearance, mice were randomized to receive either isotype or anti-mPD-1 mAb (10 mg/kg, intraperitoneally, once weekly). (B) Tumor growth curves showing ratio of tumor volume (RTV), calculated as: RTV = (tumor volume at a given time point)/(tumor volume at the initiation of treatment). Open circles represent the isotype-treated group and filled red circles represent the anti-mPD-1-treated group. (C) Tumor progression analysis in isotype- vs. anti-mPD-1-treated mice. (D) Summary data of tumor progression and survival for *Brca1^co/co^MMTV-Cre* mice treated with isotype (N = 14) or anti-mPD-1 (N = 10). (E) Western blot analysis of protein expression in tumor lysates from isotype- and anti-mPD-1-treated mice. Band densities were quantified using ImageJ and the relative expression of each antigen determined for each sample by dividing its band density by the corresponding β‑actin band density. (F) Representative histological (H&E) and immunohistochemical images (PCNA, cleaved caspase-3, CD4, CD8α, PD-1, and F4/80) of tumor sections. Scale bar: 100 μm. Data expressed as mean ± SD, and the student's *t*-test was performed (D) and (E) (*, *P* < 0.05).

To evaluate whether enhancement of the immune response can suppress *BRCA1*-associated breast cancer, we treated tumor-bearing *Brca1^co/co^MMTV-Cre* mice with anti-mPD-1 mAb or isotype antibody as a control. Spontaneous mammary tumors typically appeared from ~10 months of age, and once they reached ~0.5 cm³ in size, mice were randomly assigned into two groups receiving either isotype (N = 14) or anti-mPD-1 mAb (N = 10) intraperitoneally (i.p.) on a weekly basis (Fig. 2A). Baseline and subsequent tumor volumes were monitored until the volume approached 3 cm³. Tumor growth was quantified as the ratio of tumor volume (RTV), calculated by dividing the tumor volume at each time-point by the initial tumor volume at the start of treatment. Treatment with anti-mPD-1 mAb led to partial suppression of tumor growth compared with isotype controls, although individual variability was observed (Fig. 2B). To capture the dynamics of growth, tumor volumes measured twice weekly were converted to a measure of progression as follows: Progression (%) = (current tumor volume/previous tumor volume)×100 - 100. The distribution of these growth ratios across the treatment period is depicted in Fig. 2C. Compared with isotype controls, anti-mPD-1-treated mice showed a lower frequency of events in the higher growth categories (150-200% and >200 %) and an increased number of events in the lower categories (0-25% and 50-75%), indicating a reduced rate of tumor expansion under PD-1 blockade. The progression and survival data are summarized in Fig. 2D. N (88 for isotype and 89 for anti-mPD-1 mAb) in progression represents the total number of measurement time-points across all experimental mice. Mean ± SD values reflect the average progression over these time-points. Mice treated with anti-mPD-1 mAb exhibited a significantly lower mean tumor progression rate (58.0% vs. 105.9% in controls, *P* = 0.000087) and prolonged survival. Survival was defined as the number of weeks until the tumor volume reached 3,000 mm³, at which point mice were sacrificed following IACUC guidelines. The mean survival time was significantly longer in the anti-mPD-1 mAb cohort relative to the control group (4.45 ± 1.32 weeks vs. 3.25 ± 1.38 weeks, *P* = 0.024552).

Western blot analysis of tumor lysates showed that anti-mPD-1 treatment led to a reduction in F4/80, PD‑L1, Ki‑67, and PCNA levels, suggestive of reduced macrophage infiltration and tumor cell proliferation, although only the decrease in PCNA reached statistical significance (Fig. 2E and Supplementary Fig. 1B). Additionally, PARP expression was significantly diminished, accompanied by a downward trend in cleaved caspase‑3, supporting the theory that enhanced apoptosis serves as a contributor to delayed tumor growth. Among the signaling proteins examined, a notable decrease in phospho‑AKT was observed following anti-mPD-1 treatment, whereas phospho‑Rb, phospho‑MAPK (ERK), and phospho‑S6 levels remained unchanged, indicating that PD‑1 blockade selectively impairs the PI3K/AKT survival pathway, consistent with data obtained using a Lewis lung carcinoma mouse model 21, 22. This selective reduction in phospho-AKT is consistent with previous findings that loss of BRCA1 leads to constitutive activation of the PI3K/AKT pathway 23, 24. In contrast, MAPK and Rb signaling remain largely unaffected, even upon AKT inhibition 16. Given that PD-1 blockade can restore T cell function and reduce tumor-intrinsic AKT signaling 25, the observed decrease in pAKT likely reflects a dual effect of enhanced antitumor immunity and suppression of tumor cell survival signaling in BRCA1-deficient tumors.

IHC analysis of tumor tissues (Fig. 2F) further confirmed that anti-mPD-1 treatment led to a substantial increase in tumor‑infiltrating lymphocytes (TILs) and cleaved caspase‑3, along with moderate increases in CD4, CD8α, and PD‑1 staining coupled with a decline in F4/80, and PCNA, consistent with western blot findings. Overall, anti-mPD-1 treatment significantly delayed tumor progression and extended survival rates in *Brca1^co/co^MMTV-Cre* mice. These therapeutic outcomes were associated with increased immune cell infiltration, reduced tumor proliferation, and enhanced apoptosis within the tumor microenvironment.

### PD-1 blockade post-resection delays tumor recurrence in a model of advanced *BRCA1*-associated breast cancer

**Figure 3:**
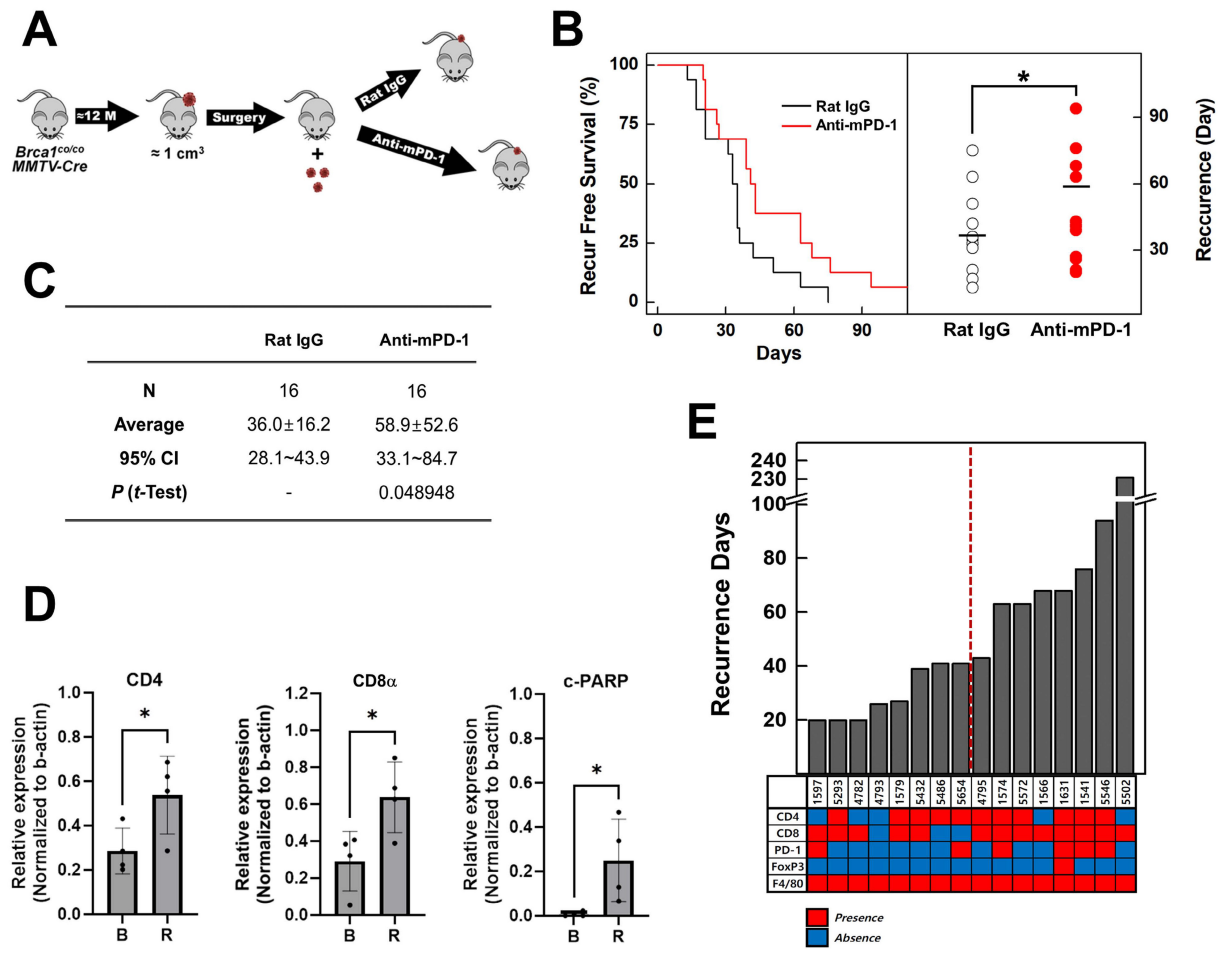
** Anti-mPD-1 mAb reduces tumor recurrence in *Brca1^co/co^MMTV-Cre* mice.** (A) Schematic diagram of the experiment. Once spontaneous mammary tumors reached a volume of ~1,000 mm³ in *Brca1^co/co^MMTV-Cre* mice, they were surgically resected. At one week post-surgery, mice were randomly assigned to receive either isotype control (rat IgG; N = 16) or anti-mPD-1 mAb (N = 16; 10 mg/kg, i.p., once/week). (B) Kaplan-Meier curves of recurrence-free survival (left panel) and scatter plot of days to recurrence (right panel) for rat IgG- (hollow circles) vs. anti-mPD-1-treated mice (red circles). (C) Summary of recurrence-free intervals. Recurrence-free periods were expressed as mean ± SD (36.0 ± 16.2 days for the isotype group and 58.9 ± 52.6 days for the anti-mPD-1 mAb group; *t*-test, *P* = 0.0489). (D) Western blot analysis. Baseline (B) and recurrent (R) tumors from rat IgG- vs. anti-mPD-1 mAb-treated mice were analyzed for the indicated immune and apoptotic markers, using β-actin as a loading control. Relative expression levels of CD4, CD8α, and cleaved PARP were calculated by normalizing the band density of each antigen to that of β-actin. (E) Days to recurrence and summary of immunohistochemical (IHC) analysis. The bar graph shows the time to tumor recurrence (Y-axis) for each mouse in the anti-mPD-1 treatment group. Heat map summarizing IHC staining for the indicated markers in the recurrent tumors. Red boxes represent the detected markers and blue boxes indicate no marker detection. Data expressed as mean ± SD, and the student's *t*-test was performed (C) and (D) (*, *P* < 0.05).

Individuals with germline *BRCA1* mutations exhibit an increased risk of breast cancer recurrence, including contralateral disease, relative to non‑carriers 26. To investigate the potential of PD-1 blockade in suppressing tumor recurrence in an advanced *BRCA1*-associated breast cancer model, we employed *Brca1^co/co^MMTV-Cre* mice that spontaneously develop mammary tumors. Tumors were allowed to progress until they reached an approximate size of 1 cm² (~1000 mm³) before surgical resection. One week after surgery, mice were randomized into two treatment groups: isotype control (rat IgG, N = 16) and anti-mPD-1 mAb (N = 16; 10 mg/kg, intraperitoneally, once per week) (Fig. 3A). Tumor recurrence was monitored over time via serial imaging and palpation. Kaplan-Meier analysis revealed that administration of anti-mPD-1 mAb significantly delayed tumor recurrence compared with the isotype control (Fig. 3B). The scatter plot of recurrence times further showed that the anti-mPD-1 mAb group displayed a shift toward longer recurrence intervals. The mean recurrence-free survival was 36.0 ± 16.2 days in the control group versus 58.9 ± 52.6 days in the anti-mPD-1 group (*P* = 0.0489) (Fig. 3C).

Western blot analysis comparing baseline (B) and recurrent (R) tumors revealed distinct changes in immune and apoptotic marker patterns (Fig. 3D & Supplemental Fig. 1C). Recurrence still occurred after anti-mPD-1 treatment, with significantly higher expression levels of CD4, CD8α, and cleaved PARP (c-PARP) in recurrent tumors compared to baseline (Fig. 3E), suggesting that the PD-1 blockade promotes an ongoing antitumor response and apoptosis. Monitoring the recurrence intervals for each mouse subjected to anti-mPD-1 therapy indicated that the recurrence times varied among individuals (Fig. 3F). The lower heat map in Fig. 3F displays IHC staining patterns in recurrent tumors (red represents positive staining and blue indicates no detection). The data suggest that mice experiencing later recurrence tend to exhibit elevated expression of T cell markers, including CD4, CD8α, and PD-1.

The collective findings suggest that enhanced T cell responses induced by anti-mPD-1 treatment are associated with delayed tumor recurrence. Anti-mPD-1 mAb treatment following surgical resection significantly prolonged the interval before tumor recurrence in *Brca1^co/co^MMTV-Cre* mice. Prolonged recurrence-free survival, was associated with increased expression of T cell markers (CD4, CD8α, and PD-1) and elevated cleaved PARP levels in recurrent tumors, indicating enhanced anti-tumor immunity and apoptosis.

### PD-1 blockade alters the lymph node immune landscape and promotes T cell activation

**Figure 4:**
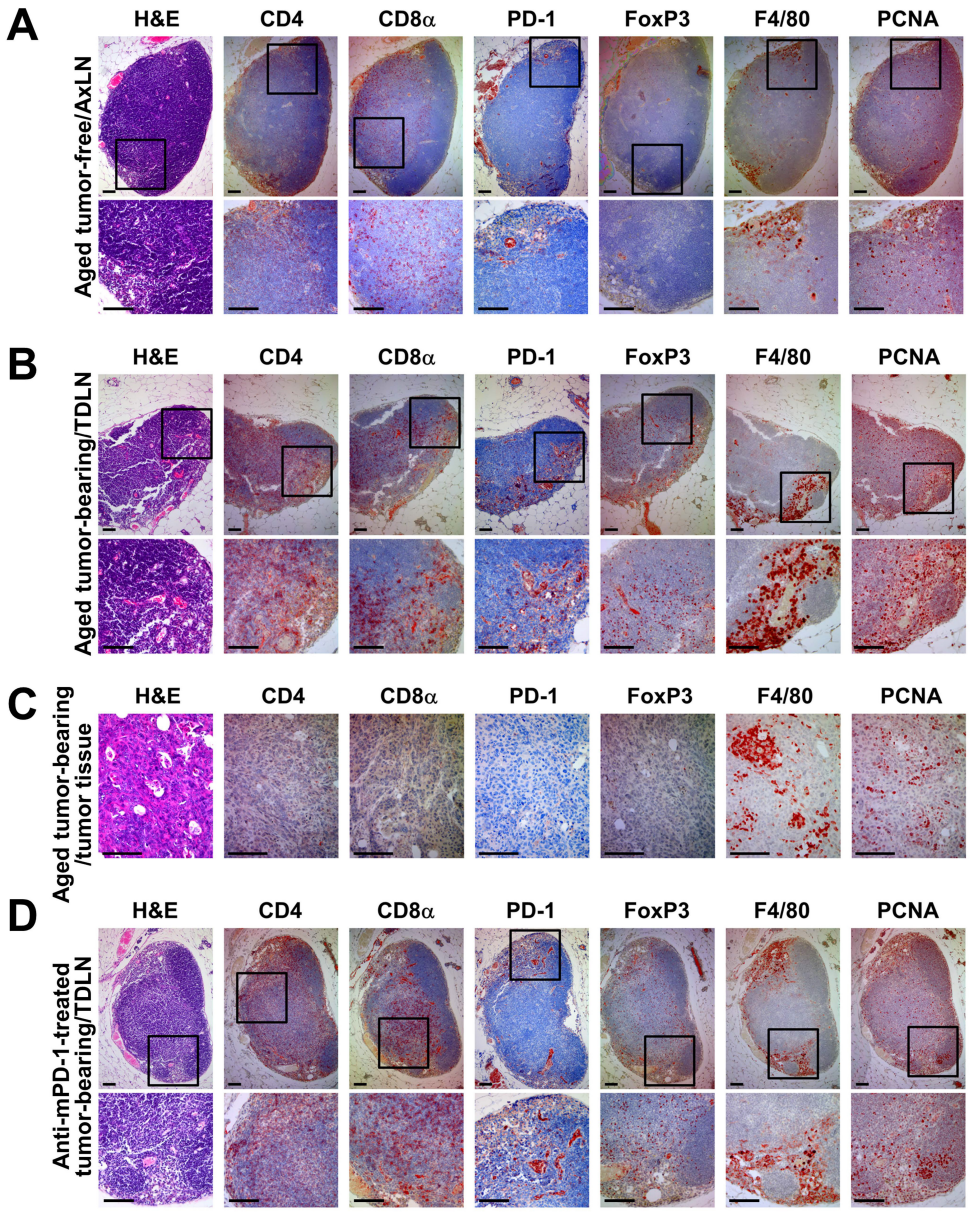
** Immunohistochemical analysis of lymph nodes and mammary tumor tissues in aged *Brca1^co/co^MMTV-Cre* mice.** (A) Representative axillary lymph node sections from an aged, tumor-free *Brca1^co/co^MMTV-Cre* mouse stained with H&E, CD4, CD8α, PD-1, FoxP3, F4/80, and PCNA. (B, C) Representative axillary tumor-draining lymph node (B) and mammary tumor tissue (C) from an aged *Brca1^co/co^MMTV-Cre* mouse with a spontaneously formed mammary tumor. (D) Representative axillary tumor-draining lymph node sections from a tumor-bearing *Brca1^co/co^MMTV-Cre* mouse treated with three doses of anti-mPD-1 mAb. In each panel, the top row shows an overview image while the bottom row provides a magnified view of the boxed region. Scale bar: 100 μm.

To further characterize the anti-tumor immune response in *Brca1*‑mutant breast cancer, we analyzed the lymph nodes, which represent the primary sites of T cell activation and priming. Immunohistochemical analysis revealed several key differences in the lymph node and tumor immune microenvironments between tumor-free, tumor-bearing, and anti-mPD-1-treated *Brca1^co/co^MMTV-Cre* mice. First, H&E staining showed that anti-mPD-1-treated mice exhibited a more structured B cell zone in the lymph nodes (Fig. 4D, arrow in the H&E‑stained panel) compared with those from tumor-free or untreated tumor-bearing mice (Fig. 4A and B), suggesting that PD-1 inhibition enhances the adaptive immune system. Second, IHC analysis of CD4 and CD8α indicated that tumor-bearing mice typically had a greater number of these T cells in lymph nodes relative to their tumor-free counterparts (Fig. 4A and B). However, their infiltration into the tumor tissue itself was not markedly increased (Fig. 4C). Treatment with anti-mPD-1 mAb led to a tendency of increased T cell proportions within lymph nodes, particularly CD8α^+^ T cells located in the T cell zone (Fig. 4D). Third, PD-1 staining was more prominent along the efferent lymphatic vessels in lymph nodes from tumor-bearing and anti-mPD-1-treated mice compared to the tumor-free control group (Fig. 2A, B, D). This finding implies that PD-1^+^ cells, potentially representing activated T cells, become more abundant in the lymph nodes following tumor-induced activation, and this increase is further enhanced by anti-mPD-1 therapy (Fig. 4D), which appears necessary for increased infiltration of activated T cells into the tumor microenvironment (Fig. 2B). Fourth, FoxP3⁺ regulatory T cells were increased in lymph nodes of both tumor-bearing and anti-mPD-1-treated mice compared to tumor-free controls (Fig. 4A, B, D), while their presence in tumor tissues was relatively low (Fig. 4C). Although anti-mPD-1 therapy did not significantly change the overall Treg proportion, CD8α⁺ and PD-1⁺ T cell populations in the lymph nodes were substantially expanded, suggesting that PD-1 inhibition may transiently relieve Treg mediated suppression, thereby enhancing the anti-tumor T cell response. Fifth, F4/80^+^ macrophages were predominantly located within the medullary sinuses of lymph nodes and further amplified by tumor growth, while their ratios remained largely unaffected by anti-mPD-1 treatment (Fig. 4A, B, D). Given that these medullary sinus macrophages are involved in the clearance of antigens and immune complexes alongside their established role in suppressing anti-tumor immune responses 23, 24, their unchanged proportion in response to anti-mPD-1 treatment suggests the requirement for additional strategies to effectively target medullary sinus macrophages for further potentiating the anti-tumor efficacy of PD-1 blockade. Finally, PCNA^+^ proliferating cells were more abundant in lymph nodes from both tumor-bearing and anti-mPD-1-treated mice compared with those from tumor-free mice, showing uniform distribution across T cell zones and marked accumulation within the medullary sinus (Fig. 4A, B, D), which implies that a substantial proportion of these proliferating cells are activated T cells.

In brief, anti-mPD-1 treatment not only enhances adaptive immunity, as reflected by increased T cell activation and proliferation in the lymph nodes, but also modulates the immune cell composition in a manner linked to delayed tumor recurrence. However, the persistence of medullary sinus macrophages suggests that further strategies to inhibit or reduce their suppressive function may be necessary to maximize the therapeutic efficacy of PD-1 blockade.

### Combined irradiation and anti-mPD‑1 mAb treatment remodels the tumor immune microenvironment in *Brca1^co/co^MMTV-Cre* mammary tumors

**Figure 5:**
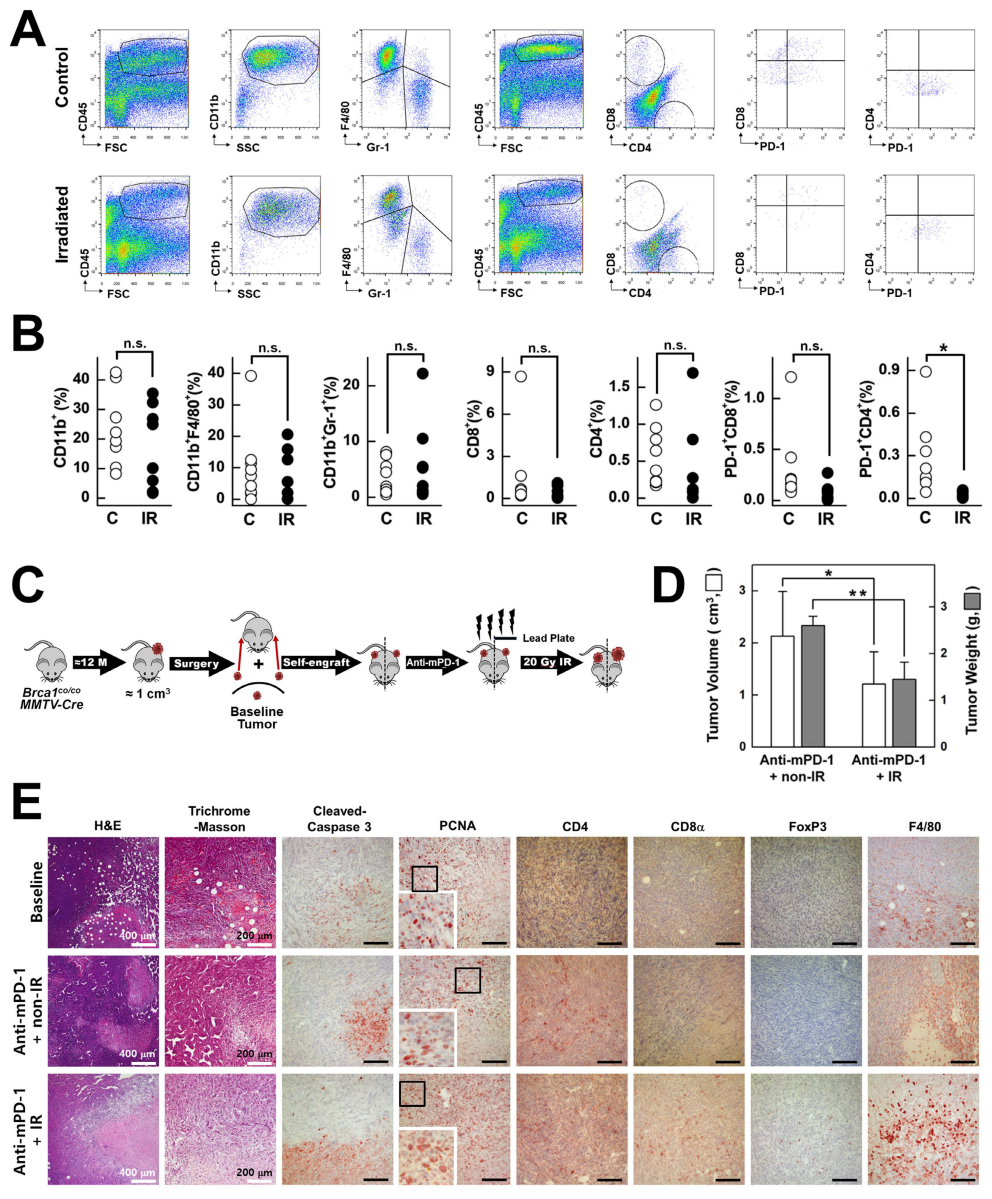
** Effects of irradiation and anti-mPD-1 mAb on the immune composition of *Brca1*-mutant mammary tumors.** (A) Representative flow cytometric analysis of control-treated (C) vs. irradiated (IR) *Brca1*-mutant mammary tumor tissues. Single-cell suspensions were prepared from tumor tissues, stained with the indicated markers (including propidium iodide, PI, to exclude dead cells), and analyzed on a FACSCalibur instrument (BD Biosciences). (B) Quantification of tumor-infiltrating leukocyte subsets in control vs. irradiated tumors from *Brca1^co/co^MMTV-Cre* mutant mice. The proportions of CD45^+^ leukocytes that were CD11b^+^ myeloid cells (further subdivided into CD11b^+^F4/80^+^ macrophages and CD11b^+^Gr‑1^+^ neutrophils), CD4^+^ T cells, CD8α^+^ T cells, and fractions of activated T cells (PD‑1^+^CD4^+^ and PD‑1^+^CD8α^+^) were calculated. (C) Overview of combined irradiation and anti-mPD-1 treatment following self-engraftment. Spontaneously formed tumors in *Brca1^co/co^MMTV-Cre* mice were surgically excised, trimmed, and re-engrafted into both sides of the mammary gland of the same mouse. At one week post‑engraftment, mice received two doses of anti-mPD‑1 monoclonal antibody for 2 weeks, and a single 20 Gy irradiation targeted to one mammary gland, with the contralateral gland protected by a lead shield. (D) Comparison of tumor volumes and weights at the endpoint between anti-mPD-1 + Non_IR-treated (N = 4) and anti-mPD-1 mAb + IR-treated tumors (N = 4). (E) Histological analyses of baseline tumors, anti-mPD-1 mAb-treated tumors, and combined anti-mPD-1 mAb/irradiation-treated tumors. Representative images depicting H&E, trichrome, cleaved caspase-3, PCNA, CD4, CD8α, FoxP3, and F4/80 staining. White Scale bars of H&E and trichrome are shown with size information in right corner of images, Black Scale bar: 100 μm. Data expressed as mean ± SD, and the student's t-test was performed (B) and (D) (*, *P* < 0.05; **, *P* < 0.05; n.s., no significance).

Local radiation is reported to reorganize the immune system, eventually leading to increased infiltration of T cells and other immune cells into the tumor microenvironment, thereby augmenting the efficacy of immunotherapy 27, 28. To determine the impact of irradiation on the tumor immune infiltrate in *Brca1^co/co^MMTV-Cre* mammary tumors, we isolated single cell suspensions from tumor tissues and conducted flow cytometry analysis of CD45^+^ leukocytes stained for CD11b, Gr 1, F4/80, CD4, CD8α, and PD-1 (Fig. 5A). Compared with non-irradiated controls, focal 20 Gy irradiation led to a widespread reduction in populations of CD11b^+^ myeloid cells, CD11b^+^F4/80^+^ tissue macrophages, CD11b^+^Gr-1^+^ neutrophils, CD4^+^ T cells, CD8α^+^ T cells, and PD-1^+^ subsets (PD-1_+_CD4^+^ and PD-1^+^CD8α^+^) (Fig. 5B). Notably, although irradiation initially induced depletion of macrophages and other leukocytes in the tumor microenvironment, the niche was rapidly repopulated by blood-derived CD11b⁺Gr-1⁻F4/80Low monocytes, leading to selective enrichment of this subset in tumor tissue (Fig. 5A; left panel). Next, to evaluate the combined effects of PD-1 blockade and irradiation, we bilaterally self-engrafted tumors into each mouse. Given the heterogeneity in characteristics and properties of tumors across individual mice, a self-engraftment strategy was used that allowed direct comparison of the therapeutic effects in identical tumors with standardization of tumor size and synchronization of treatment initiation. All animals received anti-mPD-1 monoclonal antibody treatment, and 20 Gy of focal irradiation was delivered to one tumor, while the tumor on the opposite side was shielded (Fig. 5C). Tumors treated with anti-mPD-1 in combination with irradiation (IR) showed significant reductions in both volume (43%) and weight (45%) compared to those receiving anti-mPD-1 alone (non-IR) (Fig. 5D). Masson's trichrome staining revealed a marked reduction in extracellular matrix (ECM) deposition in irradiated tumors compared to contralateral non-irradiated tumors, both under anti-PD-1 treatment, consistent with IR-induced stromal remodeling (Fig. 5E). Supporting this finding, supplemental IHC analysis further showed that irradiation reduced the abundance of cancer-associated fibroblasts (CAFs) as well as ECM components, as evidenced by decreased trichrome and CAF marker staining in irradiated tumors (Supplementary Fig. 4).

Histological analysis additionally revealed a pronounced increase in cleaved caspase 3-positive cells in the anti-mPD-1 + IR group, indicating a synergistic effect in promoting tumor cell apoptosis (Fig. 5E). Notably, PCNA staining shifted from predominantly nuclear localization in the baseline and anti-mPD-1 alone groups to a marked cytosolic distribution in tumors treated with anti-mPD-1 + IR (Fig. 5E), reflecting genotoxic stress and the involvement of PCNA in apoptosis regulation. Immunostaining further showed significant enhancement of CD8α⁺ T cell infiltration, accompanied by moderate increases in CD4⁺ T cells and FoxP3⁺ Tregs and elevated F4/80⁺ macrophage presence.

Given that IR alone led to reduced collagen deposition, increased cleaved caspase-3^+^ apoptotic cells, and reduced numbers of PCNA^+^ cells compared to non-irradiated tumors in the absence of anti-PD-1 treatment (Supplementary Fig. 5), it appears that the combination of irradiation and PD-1 blockade exerts a synergistic effect by facilitating infiltration of anti-PD-1-activated immune cells into the tumor microenvironment. This phenomenon may be mediated by IR-induced direct tumor cell killing and stromal remodeling, which collectively create a more permissive environment for immune cell access and activity.

## Discussion

In this study, we established and comprehensively characterized an autochthonous *Brca1^co/co^MMTV-Cre* mouse model that recapitulates the key features of human BRCA1‑deficient, triple‑negative breast cancer (TNBC). Our analysis revealed three interrelated mechanistic insights: (1) a prominent myeloid cell‑dominated immune barrier driven by rapid influx of blood‑derived macrophages (Fig. 1C-E), (2) capacity of the PD‑1 blockade to activate previously latent anti-tumor T cell responses and redirect PCNA toward an apoptosis regulatory role (Figs. 2-4), and (3) synergistic remodeling of the tumor microenvironment through a combination of focal irradiation and PD‑1 inhibition (Fig. 5).

During tumor progression, tumor-associated macrophages (TAMs) in this model undergo dynamic phenotypic changes, consistent with observations in other tumor models. Previous studies have demonstrated that TAMs originate from both circulating monocytes and tissue-resident macrophages, acquiring pro-tumorigenic phenotypes in response to tumor-derived cues 29, 30. For example, Franklin et al. 29 reported that mammary tumor growth promotes the accumulation of CD11b^+^MHC class II^+^ TAMs, which suppress cytotoxic T cell infiltration and thereby promote tumor progression. Consistent with these findings, we observed a similar phenotype of tumor macrophages in tumor tissues of *Brca1^co/co^MMTV-Cre* mice (Supplementary Fig. 6), suggesting their potential involvement in promoting tumor growth. However, given the complexity of TAM-mediated tumor progression (including secretion of growth factors, promotion of angiogenesis, remodeling of the extracellular matrix, and suppression of cytotoxic T cell activity), the precise mechanisms underlying their contribution in this model will be further investigated in a separate study.

In the context of current clinical standards of care for TNBC, particularly combinations of DNA‑damaging agents, PARP inhibitors, and immunotherapies, our findings underscore the significant translational relevance of this model and justify its suitability as a preclinical platform for evaluating novel TNBC regimens. Flow cytometry experiments revealed that during the early stages of tumor development, the macrophage compartment in *Brca1^co/co^MMTV-Cre* lesions was dominated by CD11b⁺Gr‑1⁻F4/80^Low^ blood‑derived macrophages, accounting for nearly 90% of CD11b⁺Gr‑1⁻ cells (Fig. 1C). However, once tumors were fully established, the balance shifted in favor of CD11b⁺Gr‑1⁻F4/80^High^ tissue‑resident macrophages (Fig. 5A), consistent with reports that circulating monocytes progressively acquire a tissue phenotype within the tumor microenvironment 29, 30. Importantly, the maturation dynamics of macrophages was further modulated by focal irradiation and PD‑1 blockade, which reshaped the overall myeloid landscape in treated tumors (Fig. 2F, 3D, 5A, and 5E). Medullary sinus macrophages (MSMs) in the draining lymph nodes similarly remained abundant after the administration of anti-mPD‑1 therapy (Fig. 4A‑D). MSMs are recognized for their ability to clear antigens and immune complexes, along with secretion of immunosuppressive cytokines that physically restrict lymphocyte migration into tumors 31, 32. Their prevalence in both tumor and nodal lymph node sites suggests that macrophage‑mediated suppression is a major barrier to effective antitumor immunity in BRCAness TNBC.

Despite the low baseline abundance of CD4⁺ and CD8α⁺ T cells in tumors (<1% of CD45⁺ cells; Fig. 1C), treatment with anti‑mPD‑1 mAb significantly delayed both tumor progression and recurrence (Figs. 2, 3). Western blot analysis revealed that PD‑1 blockade led to a specific notable decrease in phospho‑AKT among several survival pathways, highlighting selective impairment of PI3K/AKT signaling (Fig. 2E) 21, 22. Concurrently, markers of proliferation (PCNA, Ki‑67) and macrophage infiltration (F4/80) were decreased whereas cleaved PARP levels showed an upward trend, reflecting enhanced apoptosis. Immunohistochemical analysis confirmed an increase in TILs, in particular, PD‑1⁺CD8α ⁺ cells (Fig. 2F). Moreover, in the post‑resection recurrence model, recurrent tumors exhibited markedly higher CD4, CD8α, and cleaved PARP levels than baseline (Fig. 3E). Additionally, mice with delayed recurrence displayed stronger CD4, CD8α, and PD‑1 staining in both their tumors and draining lymph nodes (Fig. 3F). The results collectively demonstrate that PD‑1 blockade reinvigorates exhausted T cells, promotes apoptotic engagement of PCNA, and induces durable antitumor activity, even in an environment dominated by suppressive myeloid cells.

We additionally exploited the “damage‑plus‑immune” paradigm by combining focal 20 Gy irradiation with PD‑1 blockade in self‑engrafted tumors. Radiation alone depleted the majority of leukocyte subsets but paradoxically triggered rapid repopulation by CD11b⁺Gr‑1⁻F4/80^Low^ monocytes, indicating that radiation creates an immunological “niche” primed for monocyte infiltration (Fig. 5A‑B). When used in conjunction with anti‑mPD‑1 mAb, irradiation induced a synergistic increase in cleaved caspase‑3 (indicative of apoptosis) and shift in PCNA staining from nuclear (proliferation) to cytosolic distribution (stress‑ and apoptosis‑regulated) (Fig. 5E). Additionally, Masson's tric**h**rome staining revealed a reduction in extracellular matrix deposition, consistent with stromal remodeling (Fig. 5E). This ECM reduction was primarily attributed to irradiation-induced depletion of cancer-associated fibroblasts (CAFs), which are the main source of stromal matrix proteins (Supplementary Fig. 4). CAF depletion effectively altered the dense stromal barrier, transiently facilitating immune cell infiltration. As a result, the tumor microenvironment became more conducive to the entry and activation of effector T cells, thereby amplifying the therapeutic efficacy of PD-1 blockade. This dual treatment markedly amplified CD8α⁺ T cell infiltration and led to a moderate increase in CD4⁺ T cells, Tregs, and macrophages, indicating an orchestrated inflammatory influx that overrides baseline immunosuppression. Together, these findings highlight the mechanistic complementarity of DNA damage and immune checkpoint inhibition in reshaping the tumor microenvironment.

Current TNBC treatment strategies in the clinic reflect a similar “damage‑plus‑immune” approach. In early‑stage TNBC, standard neoadjuvant regimens typically consist of anthracycline‑taxane combinations with or without carboplatin, which induce DNA damage and promote immunogenic cell death 33. The KEYNOTE‑522 trial further demonstrated that the incorporation of pembrolizumab (an anti-mPD-1 agent) into neoadjuvant chemotherapy significantly improved pathologic complete response rates 34, validating the concept of combining cytotoxic damage with immunotherapy. Moreover, for patients with germline *BRCA1* or *BRCA2* mutations, adjuvant PARP inhibitors, such as olaparib (OlympiA trial), exploit homologous recombination deficiency to generate lethal DNA lesions, an approach analogous to our use of irradiation in a *Brca1* knockout context 35, 36. In the metastatic setting, the efficacy of PD‑L1 inhibitors in combination with nab‑paclitaxel and antibody-drug conjugates, such as Sacituzumab govitecan, further underscores the necessity for preclinical models that accurately replicate both the genetic vulnerabilities and immunologic milieu of human TNBC 37, 38.

The *Brca1^co/co^MMTV-Cre* model offers a uniquely relevant in vivo platform for preclinical drug development by effectively reproducing the key hallmarks of human *BRCA1*-associated mammary tumors, including basal-like histology, dominant blood-derived macrophage infiltration, low baseline TIL density, and sensitivity to both DNA damage and immunotherapy. Future studies should focus on leveraging this model to: (1) assess macrophage-targeting agents (e.g., CSF-1R inhibitors) in combination with PD-1 blockade, (2) evaluate novel DNA damage response inhibitors alongside immunotherapeutic strategies, and (3) determine the spatiotemporal dynamics of PCNA relocalization and its functional crosstalk with apoptotic and repair pathways. Consistent with other spontaneous breast cancer models such as MMTV-NeuT and MMTV-PyMT 39, 40, anti-PD-1 monotherapy in *Brca1^co/co^MMTV-Cre* mice induced only partial tumor suppression (Figs. 2B-D, 3B-C). Although these models represent distinct breast cancer subtypes, they share a highly immunosuppressive tumor microenvironment that limits complete tumor regression by immune checkpoint blockade alone 39, 40. Notably, the *Brca1^co/co^MMTV-Cre* model closely reflects the clinical features of TNBC, where immunotherapy alone rarely achieves cure 41-43, supporting its translational relevance for evaluating combination strategies designed to overcome this resistance.

In conclusion, our data provide a mechanistic framework for overcoming the myeloid‑mediated immune barrier and desmoplasia in TNBC through a combination of damage and immune strategies. The translational alignment of our preclinical findings with existing and emerging clinical paradigms positions the *Brca1^co/co^MMTV-Cre* mouse as an optimal platform for accelerated evaluation of next‑generation combination therapies targeting BRCA1‑deficient breast cancer. Future studies will further leverage this model to dissect the cellular and molecular mechanisms that promote or restrain tumor progression, with particular emphasis on functional analyses of CD8^+^ T cells, PD-1^+^ macrophages, and other immune and stromal components within the tumor microenvironment.

## Supplementary Material

Supplementary figures.
