# Supplementary material for: Synergistic Effects of Radiotherapy and PD‑1 Blockade in a Human‑Mimetic BRCAness Model of Triple-Negative Breast Cancer
Source: Int J Biol Sci. 2025 Oct 1;21(14):6270–84. doi: 10.7150/ijbs.118427 (PMC12594595; doi:10.7150/ijbs.118427)
Supplement: Supplementary file 1 — Supplementary figures. [file ijbsv21p6270s1.pdf]

Supplemental Information

Synergistic Effects of Radiotherapy and PD-1 Blockade in a Human-Mimetic BRCAness Model of Triple-Negative Breast Cancer

Eun Ju Cho, Min Kyung Ki, Hye Jung Baek, Dong Hoon Shin, Eun Jung Park, Tae Hyun Kim, Chu-Xia Deng, Beom K. Choi, and, Sang Soo Kim

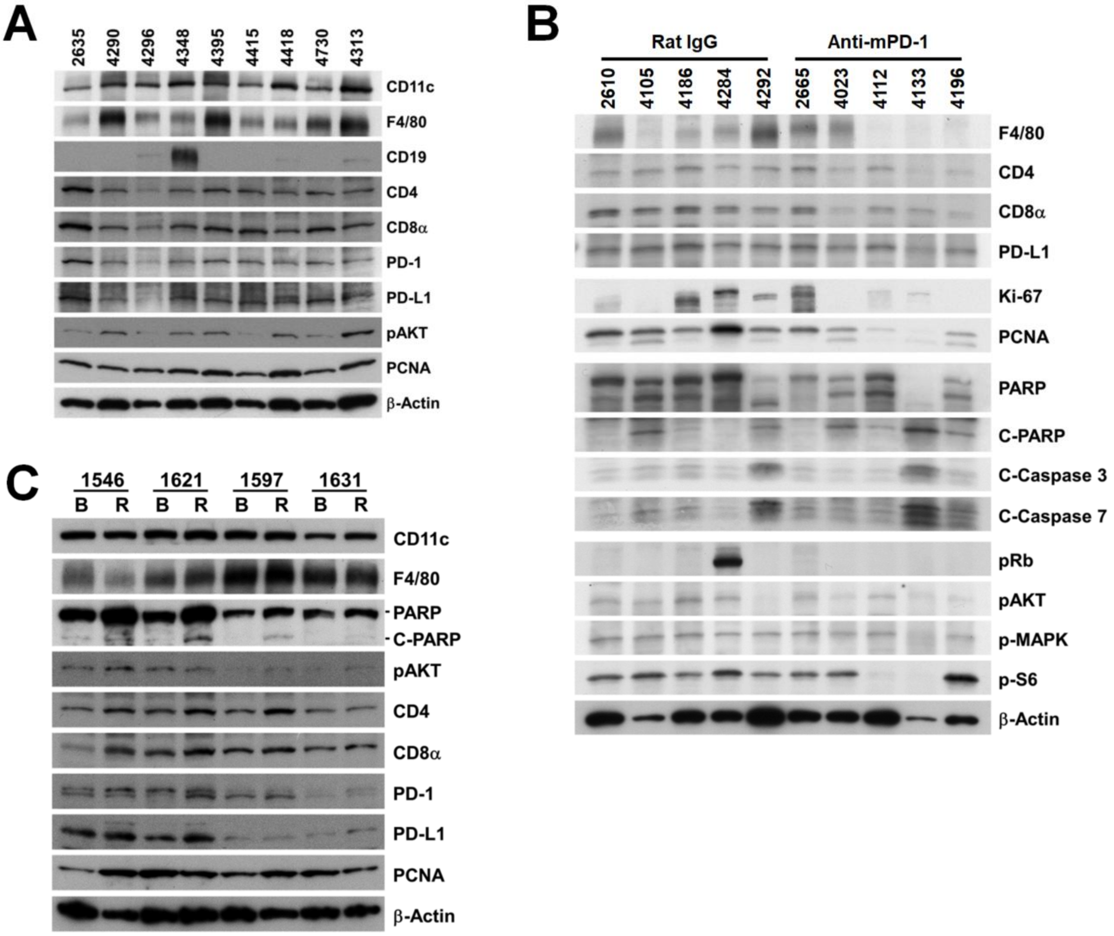

Supplementary Figure 1. Western blot analysis of the expressed proteins in *Brca1$^{co/co}$MMTV-Cre* mammary tumors. (A) Analysis of the immune-related proteins in spontaneously developed mouse mammary tumors. (B) Analysis of protein expression in tumor lysates from isotype- and anti-mPD-1-treated mice. (C) Baseline (B) and recurrent (R) tumors from rat IgG- vs. anti-mPD-1 mAb-treated mice were analyzed for the indicated immune and apoptotic markers. β-actin was used as a loading control. The numbers above each lane indicate individual mouse identification numbers.

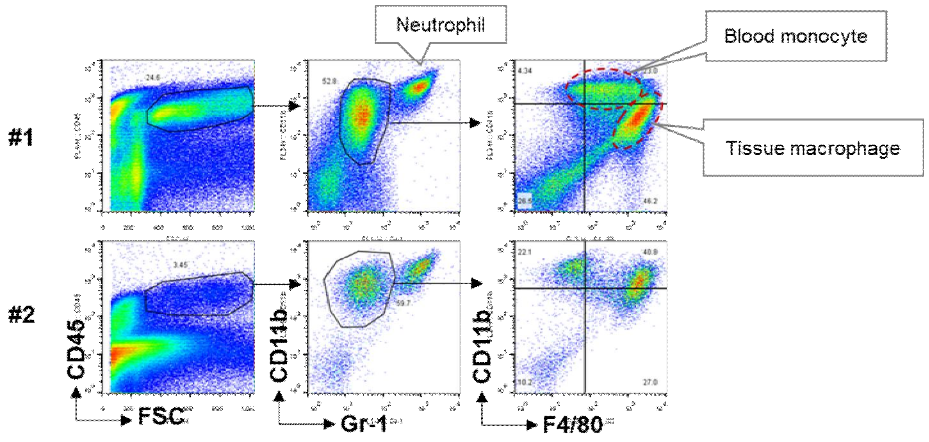

Supplementary Figure 2. Flow cytometric analysis of tumor-infiltrating myeloid cells. Single-cell suspensions were prepared from tumor tissues and stained with anti–CD45-APC, anti–Gr-1-FITC, anti–CD11b-PE-Cy5, and anti–F4/80-PE antibodies. Samples were acquired using a FACSLyric flow cytometer (BD Biosciences). Myeloid cells were initially identified by gating on $CD45^{+}$ leukocytes, followed by selection of $CD11b^{+}Gr\text{-}1^{Low}$ cells. These cells were subsequently analyzed by plotting F4/80 versus CD11b expression to distinguish blood-derived ($F4/80^{Low}CD11b^{High}$) from tissue-resident ($F4/80^{High}CD11b^{Low}$) macrophage populations.

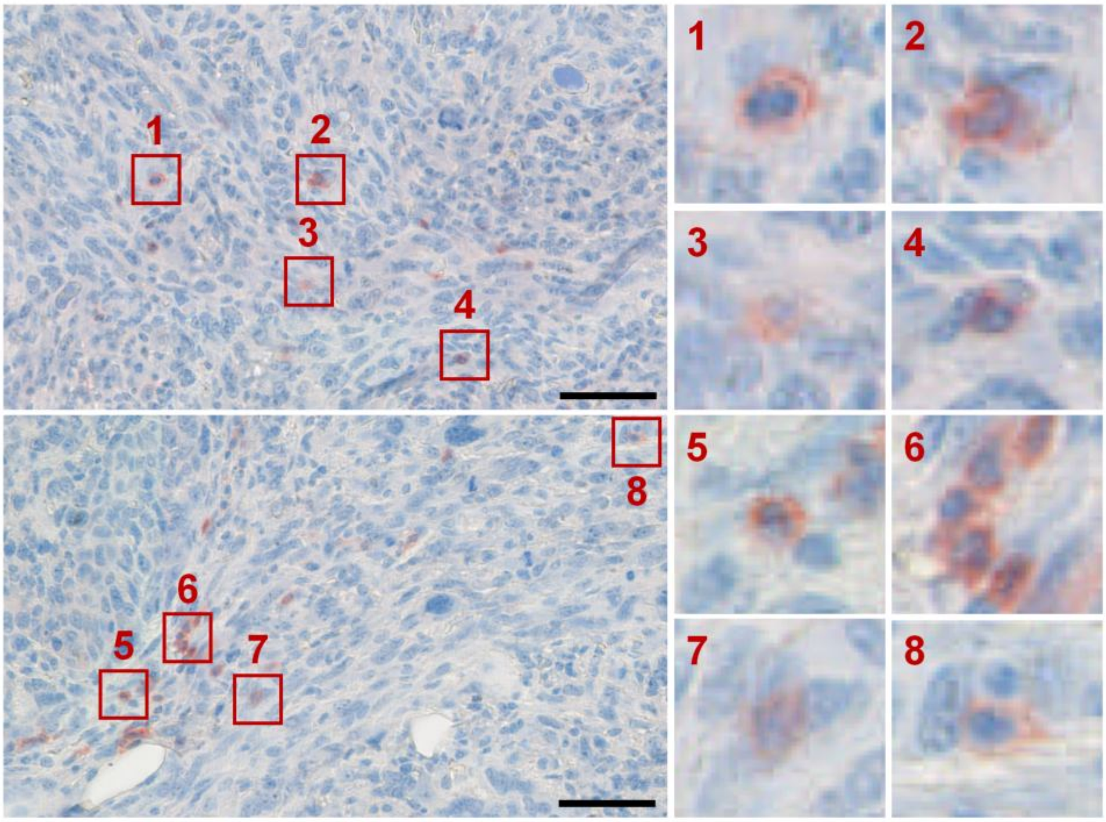

Supplementary Figure 3. Higher-magnification images of PD-1 immunohistochemical staining in mammary tumor sections. Mammary tumor sections were stained with an anti–PD-1 antibody. The numbered boxed areas in the left images are enlarged in the corresponding right panels. Scale bar: 50 µm.

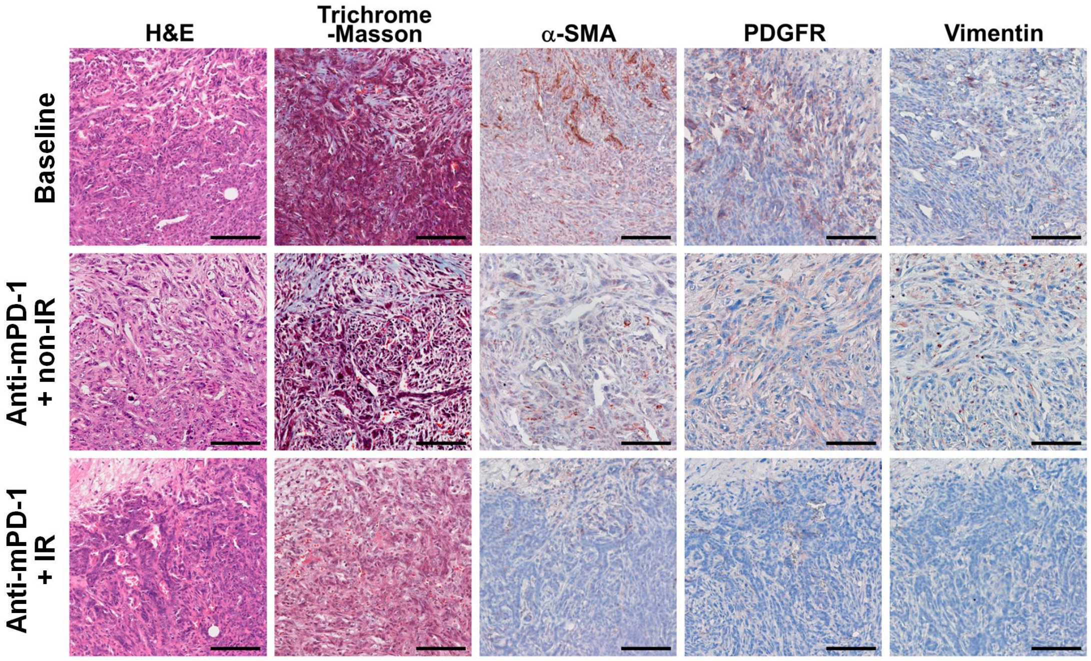

Supplementary Figure 4. Effects of irradiation on stromal remodeling in anti–mPD-1 mAb treated BRCA1-deficient mammary tumors. Histological analyses of baseline tumors, anti-mPD-1 mAb-treated tumors, and combined anti-mPD-1 mAb/irradiation–treated tumors using antibodies targeting cancer-associated fibroblasts. Scale bar: 100 μm.

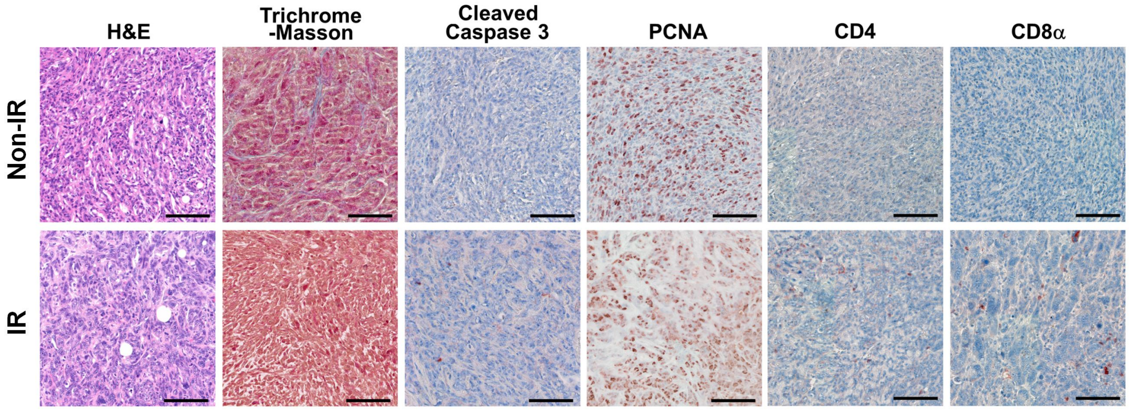

Supplementary Figure 5. Effects of irradiation on immune cell infiltration in BRCA1-deficient mammary tumors. Spontaneously formed tumors in *Brca1*$^{co/co}$*MMTV-Cre* mice were surgically excised, trimmed, and re-engrafted into both sides of the mammary gland of the same mouse. At one week post engraftment, mice received a single 20 Gy irradiation targeted to one mammary gland, with the contralateral gland protected by a lead shield. Representative images of the histological features of non-irradiated (non-IR) and irradiated (IR) tumors. Tumor sections were stained with H&E for histological analysis, Masson's trichrome for assessment of collagen deposition, and subjected to immunohistochemistry for cleaved caspase-3, PCNA, CD4, and CD8α. Scale bar: 100 μm.

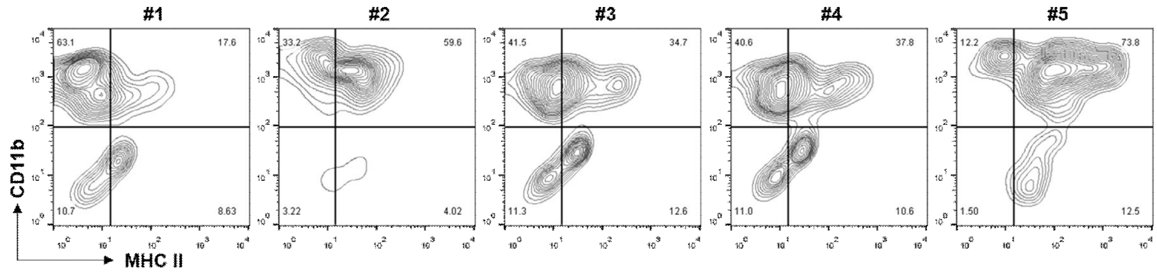

Supplementary Figure 6. Flow cytometry analysis of macrophage populations in mammary tumors. Tumor tissues were harvested from five individual mice bearing tumors of varying sizes. Single-cell suspensions were prepared from tumor tissues, stained with anti-CD45-APC, anti-CD11b-PE, and anti-MHC class II-FITC antibodies, and analyzed using flow cytometry. Myeloid cells were identified by initial gating on $CD45^+$ leukocytes, followed by analysis of CD11b and MHC class II expression.

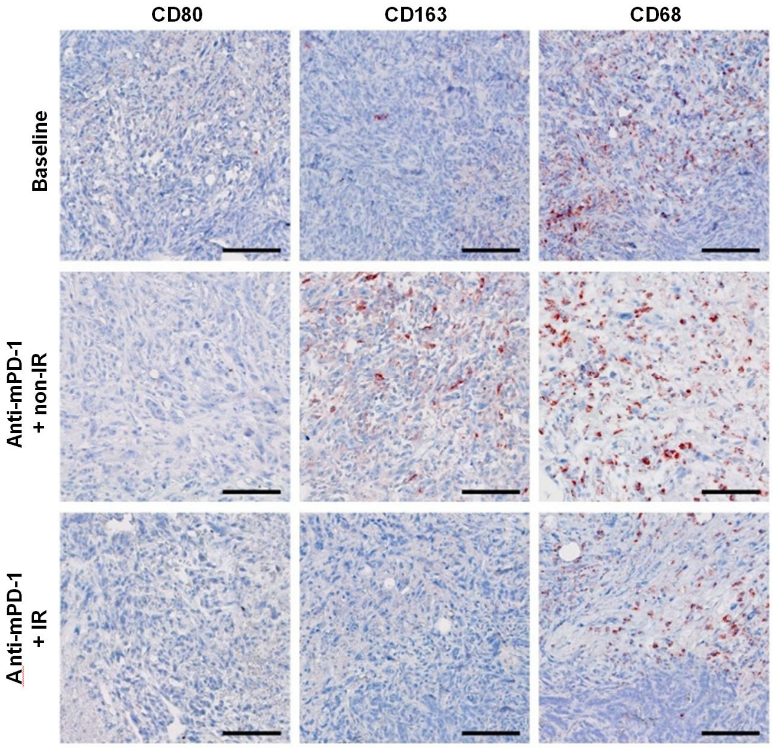

Supplementary Figure 7. Immunohistochemical analysis of tumor-associated macrophage markers CD80, CD163, and CD68 in baseline and self-engrafted tumor tissues. Tumors were implanted by subcutaneous self-engraftment into both flanks of mice. Following engraftment, mice were treated with antibiotics for 7 days, followed by intraperitoneal administration of anti-mPD-1 antibody twice over two weeks. Focal irradiation (IR) was subsequently applied to one of the tumors. "Baseline" indicates tumor tissues collected in surgery for engraftment. "Anti-mPD-1 + non IR" indicates non-irradiated tumors from mice treated with anti-PD-1 antibody. "Anti-mPD-1 + IR" indicates irradiated tumors from the same mice. IHC staining was performed on formalin-fixed, paraffin-embedded tumor sections using antibodies specific for CD80 (M1 marker), CD163 (M2 marker), and CD68 (pan-macrophage marker). Scale bar: 100 μm.
